# Radiation effect or siphon effect? Coupled coordination and spatial effects of digital economy and green manufacturing efficiency—Evidence from spatial Durbin modelling

**DOI:** 10.1371/journal.pone.0313654

**Published:** 2024-11-14

**Authors:** Xiaoshu Sun, Wanyu Zhang, Xianming Kuang

**Affiliations:** 1 School of Business Administration, Northeastern University, Shenyang, China; 2 China Institute for Reform and Development, Haikou, China; Sichuan University, CHINA

## Abstract

This study uses the non-expected SBM-DEM model of factor input-output to measure the green manufacturing efficiency of 274 cities at prefecture level and above in China. The relationship between digital economy and green manufacturing efficiency is analysed on this basis. It is found that the current coupling coordination between digital economy and green manufacturing efficiency is low overall, and there are obvious regional differences. And the digital economy has a significant positive impact on the green manufacturing efficiency of the region and the neighbouring regions, so the digital economy has a radiation effect on the green manufacturing efficiency. Among them, industrial agglomeration plays part of the mediating effect. Based on this conclusion, the following policy recommendations are proposed: first, accelerate the development of the digital economy to stimulate the new kinetic energy of urban green development. Second, implement differentiated development strategies, focusing on the leading role of green advanced cities. Third, deepen the convergence of industries and smooth the channels for green urban development.

## 1. Introduction

The emphasis of China’s development has increasingly moved from speed to quality as the country’s economic development level rises. When people’s requirements are progressively unmet by careless development and blind expansion, the value of environmental preservation and resource conservation grows. The manufacturing sector in China, which is the backbone of the country’s economic growth, is still far from reaching a level of high-quality development that balances environmental preservation with economic growth, and there is still a clear conflict between the increased pollution that comes from manufacturing production and the advancement of manufacturing efficiency. High levels of pollution have always accompanied its rapid rate of growth. China is ranked 120th out of 180 nations in the 2020 Global Environmental Performance Index [1], with a 56.7% coal consumption share. Fossil energy continues to dominate global energy consumption, making environmental challenges a major roadblock to China’s high-quality development. Reduced green manufacturing efficiency is now a significant barrier to China’s transition to a sustainable development model and green economy.

The digital economy has emerged as a key strategy for reducing the strain on resources and the environment in light of the shared requirements of resource scarcity and environmental conservation. Digital technologies, including big data and the Internet, are essential to the process of moving from undeveloped to developed states. The digital economy is also seen as a key strategic development direction by many nations worldwide. The scope economy, scale economy, and long-tail effect of the digital economy can aid in removing the barrier of resource constraints and encourage the green transformation of industrial businesses. It will be possible to support the green development of the industrial sector and raise the bar for sustainable development by integrating the digital economy and manufacturing sector to accomplish the digitalization and intelligence of the manufacturing sector.

There are natural variances in the level of growth of the digital economy between regions due to differences in infrastructure conditions. It is probable that the exceptional advancement of the digital economy in one of the nearby regions would radiate outward, resulting in the shared progress of the neighboring regions. On the other hand, as the digital economy grows, so does the phenomenon of industrial agglomeration, and knowledge and technology tend to concentrate more and more in the same region. This has a siphoning effect on neighboring industries, leading to detrimental effects on the economy and environment as well as a widening of interregional differences [2].

Thus, investigating the connection between the growth of the digital economy and the effectiveness of green manufacturing is crucial. Because of this, the research object of this paper is the digital economy and green manufacturing efficiency. The super-efficient SBM-DEA model is used to calculate the green manufacturing efficiency while accounting for non-desired output. The correlation between the two variables, particularly the spatial correlation, is then analyzed in an effort to support future cooperation amongst Chinese regions in pursuit of the "dual-carbon" goal. The results indicate that the performance of the region with respect to the "dual-carbon" target is significantly influenced by the digital economy. Based on the findings, it can be concluded that the digital economy has a significant positive impact on the green manufacturing efficiency of the local area and a significant negative impact on the green manufacturing efficiency of neighboring regions. And modernizing the industrial structure is a key mediating factor in it.

The marginal contributions of this study are mostly in the following three aspects. Firstly, by incorporating earlier research, this paper creates a more lucid assessment index system for the efficiency of green manufacturing and the digital economy. The computation indicates that green production efficiency is still poor at this point. Secondly, this paper constructs a coupling coordination degree measurement model to measure the coupling coordination degree between digital economy and green manufacturing efficiency and to analyse regional differences. Third, this study creates a spatial econometric model to evaluate the geographical relationship between the digital economy and green manufacturing efficiency, which adds to the current debate on spatial relationships. The efficiency of green manufacturing is found to be positively impacted by the digital economy locally, but negatively impacted by it in neighboring regions. Lastly, the mediating role of industrial agglomeration is the main topic of this article, which also reveals the precise mechanism path of the digital economy influencing the efficiency of green manufacturing. It is discovered that the influence of the digital economy on the efficiency of green manufacturing in neighboring regions is partially mediated by industrial agglomeration.

The subsequent sections of this paper are delineated as follows: Part II furnishes a comprehensive literature review, Part III expounds upon the theoretical analysis and research hypotheses, Part IV succinctly outlines the data sources employed in this study alongside the research methodology, Part V engages in an analytical discourse concerning the findings, and Part VI culminates by presenting key conclusions and offering policy recommendations.

## 2. Literature review

A growing number of academics have focused increasingly on high-quality development in recent years as a result of mounting resource limitations and environmental demands. As a crucial component of superior development, green productivity research has gained a lot of attention. Its primary focus is on quantifying green productivity and identifying the variables that impact it.

Green production efficiency has been measured using a variety of techniques, including single-indicator measurement, parametric methods represented by stochastic frontier analysis (SFA) [3], non-parametric methods using data envelopment analysis (DEA) [4], and the Super-SBM model [5]. The progression of the By including non-desired outputs into the Super-SBM model, a more accurate estimate of green production efficiency can be obtained by optimizing efficiency while limiting environmental damage [6, 7]. As a result, this study will also use this methodology to calculate the efficiency of green production.

The following are some of the factors that influence the efficiency of green production: economic growth [8]; technological level; energy consumption structure [9]; industrial structure; degree of urbanization [10]; and environmental regulation [11]. Strict policy enforcement, in particular, can encourage technical advancement and contribute to green productivity in manufacturing.

In the current booming development of digital economy, data as a new production element plays a vital role in enhancing production efficiency, optimizing resource allocation and encouraging green and sustainable development [12]. There are very few studies on the production efficiency of other industries, with the majority of current research focusing on the green production efficiency of agriculture [13, 14]. Nonetheless, a number of studies have already discovered that the growth of the digital economy would encourage an increase in the effectiveness of green innovation [15]. However, this effect is non-linear, with a diminishing tendency as the level of digital economy develops [16] and there is a threshold effect. The development of a region’s digital economy will have spatial spillover effects on associated regions, although the digital economy centered on data elements has the inherent benefit of transcending geographical distance and spatial limits [17].

The geographical relationship between the digital economy and green productivity in manufacturing has already been examined in certain publications. On the other hand, there hasn’t been a clear consensus in the literature to suggest whether there is a positive spillover effect or a negative siphoning effect. According to some experts, the growth of the digital economy will speed up technological transfer and have a demonstrative influence on nearby areas, resulting in a positive radiation effect [18, 19]. There is a school of thought that holds that as the digital economy grows, it will attract production elements from nearby areas, so reducing the area available for green growth in those areas and creating a siphoning effect [20]. Consequently, in order to determine whether the geographic influence of the digital economy on green manufacturing efficiency is a siphon effect or a radiation effect, a more thorough investigation of this matter is required.

In conclusion, this article has been scrutinized utilizing data from Chinese towns in order to further analyze the lines of impact and spatial correlations that exist between the digital economy and the efficiency of green manufacturing.

## 3. Theoretical mechanisms

The shift of the old economic development mode is significant with the advent of the digital economy. Many new sectors and business models have grown as a result of the expansion of the digital economy, and new economic models like the sharing economy, platform economy, online office, virtual industrial park, and industrial cluster have also evolved. The increased acceptance of the sharing economy and platform economy can lead to better resource and equipment sharing, resource transformation, full resource utilization, and ultimately reduced resource waste to improve green efficiency. At the same time, the development of digital economy means the continuous improvement of digital infrastructure. The upgrade of equipment will bring the simultaneous upgrade of technology level, thus guiding enterprises to transform to clean production, fully applying clean production process and technology to all aspects of traditional manufacturing industry, eliminating backward production technology and backward production capacity, and guiding manufacturing industry to develop in a greener direction [21].

Unavoidably, a wider usage of digital components follows the growth of the digital economy. The free flow of digital materials will, on the one hand, make it easier to realize information interchange and communication, increasing business relationships and bringing about improvements in green efficiency. On the other hand, it will improve the links between various sectors, strengthen the close collaboration between the upstream and downstream of the industrial chain, realize the specialization and refinement of each sector through the industrial correlation effect, achieve the green development of resource conservation, and boost the effectiveness of green manufacturing. In contrast to conventional resources, data elements have the benefit of being easier to duplicate and share. It can be viewed as a lower cost, greater return element of production since it has a higher marginal utility during usage and a marginal cost that is near to zero. As a result, in the digital economy model, producers can employ digital resources to get more economic benefits from using less natural resources, which ultimately leads to reduced pollution. This supports the following theory:

H1: The digital economy will help promote green manufacturing efficiency.

When considered from a spatial viewpoint, data has a natural tendency to move, increasing market transparency and hastening the diffusion of knowledge and information. It can also overcome physical and temporal borders, weakening conventional interregional boundaries. For instance, the barriers resulting from physical distance between different businesses have been weakened by the Internet’s rapid expansion as a key component of the digital economy. As a result, it is simpler and more effective for businesses to copy one another and learn from one another from surrounding locations where they have comparative advantages [22]. As a result, the growth of the digital economy naturally has an impact on the neighborhood.

Manufacturing businesses in many regions are able to rely on digital components for greater cross-regional cooperation against the backdrop of the growing relevance of green sustainable development. The goal of swiftly disseminating experiences connected to green development can be achieved by expediting the flow of green production elements in the manufacturing industry between regions. Additionally, because the environmental challenges faced by businesses in similar spatial and geographic locations will be more comparable, it will be simpler to draw lessons from one another’s experiences. A phenomenon known as "competition by competition" may arise among cities as a result of the development of the digital economy in the central city [23]. This phenomenon encourages industrial enterprises in neighboring cities to compete with one another continuously, improving the efficiency of green manufacturing in each location while also promoting the efficiency of green manufacturing in nearby areas through demons. Through demonstration and warning effects, it will also simultaneously encourage green manufacturing efficiency in the surrounding areas. This leads to the hypothesis that,

H2a: There is a spatial spillover effect of the digital warp economy on the green manufacturing efficiency of neighboring cities.

On the other hand, however, constrained by the unequal distribution of information resources, the development of the digital economy may result in an economic Matthew effect, which would cause the agglomeration effect of economic development levels to increase and eventually form an economic geographical pattern that tends to agglomerate [24]. In terms of the green economy, this is demonstrated by locations with higher levels of digital economic development having the capacity and propensity to move highly polluting manufacturing industries to nearby regions in order to attain local green manufacturing efficiency. Because the regions that passively receive industries frequently have not yet reached the inflection point of the environmental Kuznets curve, the short-term development of the digital economy and manufacturing will further intensify the consumption of resources and energy, raising pollution levels [25]. At the same time, the growth of the digital economy will worsen digital technology’s capacity to deplete and devour resources in the absence of good leadership. The price mechanism lowers the cost of natural resources while increasing the reliance of profit-seeking corporate organizations on those resources. In other words, the growth of the digital economy makes it easier to access resources, enhances the availability of resources, lowers the cost of resources, and further solidifies the dominance of natural resources. Eventually, the preference for capital, skills, and other criteria will supplant the preference for natural resources, reducing the efficiency of green manufacturing even further. This supports the opposing theory that:

H2b: There is a negative spatial spillover effect of the digital warp economy on the green manufacturing efficiency of neighboring cities.

**Fig 1:**
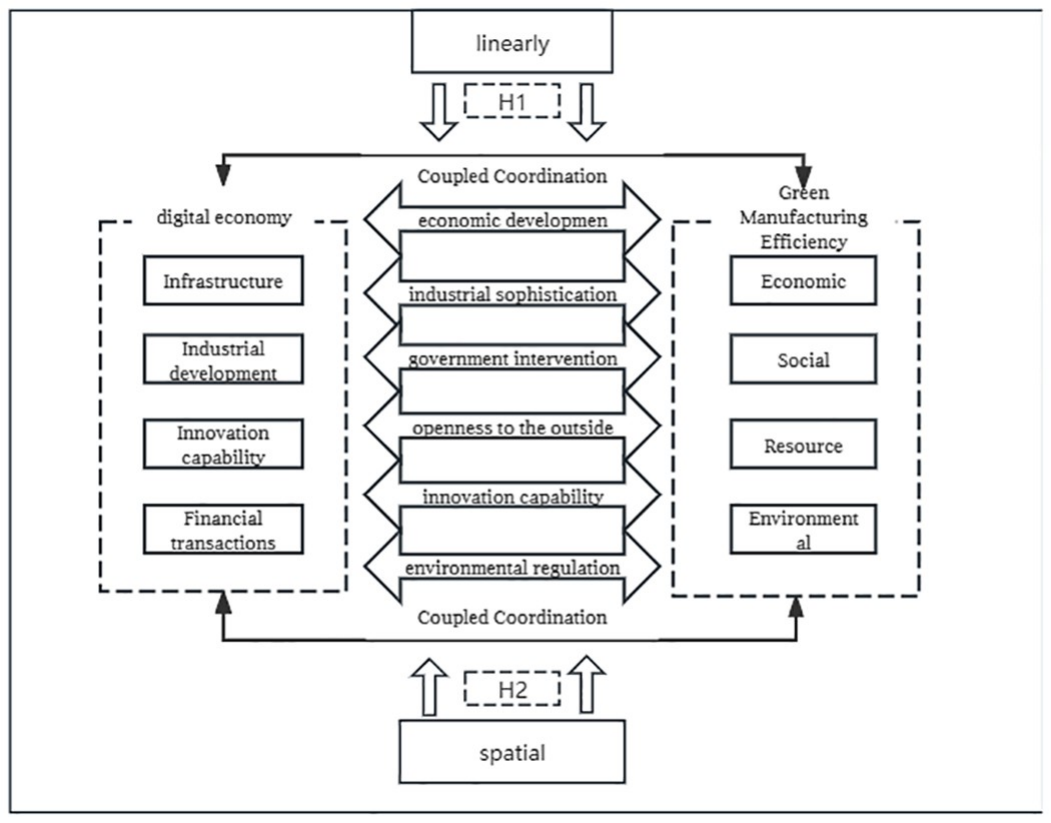
Mechanisms of influence.

The mechanism of influence is shown in Fig 1.

In the process of digital economy development, it often attracts neighbouring industries to gather in the same region through the siphon effect, forming the agglomeration effect of industries [26]. With the agglomeration of industries, a series of new modes and new business forms will be generated, accelerating the change of production and life style. The industrial agglomeration brought about by the development of the digital economy will have spatial spillover effects on the neighbouring regions through the trickle-down effect, the learning effect and the sharing effect [27]. On the one hand, industrial agglomeration will help enterprises to share the corresponding infrastructure and improve the utilisation of resources. On the other hand, it will make it easier for enterprises to imitate and cooperate with each other, accelerate the formation of green production atmosphere, and ultimately promote the synergistic improvement of green manufacturing efficiency.

H3: Industrial agglomeration plays a mediating role in the spatial effect of digital economy on green manufacturing efficiency.

## 4. Model construction and data

### 4.1 Spatial Durbin model

Considering that the spatial Durbin model is able to measure the impact on local and neighbouring regions at the same time, this paper adopts the spatial Durbin to analyse the spatial dependence between the digital economy and green manufacturing efficiency, from which the model of this paper is constructed:

Yit=α+0ρWYit+α1A+itα2WAit+γ1Xit+γ2WXit+μi+η+tδit

Where W is the spatial weight matrix, and in this study spatial distance W_d_, economic distance W_e_ and nested matrix W_ed_ considering economic and geospatial distances are used. Y_it_ denotes the explanatory variable green manufacturing efficiency, ρ denotes the estimated coefficient of spatial regression. A_it_ denotes the explanatory variable digital economy, and X_it_ denotes other control variables that would cause effects. μ denotes provincial fixed effects, η denotes time fixed effects, and δ denotes the random disturbance term.

In the process of analysing spatial measures, the following three spatial weighting matrices are selected for analysis in this paper.

#### (1) Geographic distance spatial weight matrix (W_d_)

According to the first law of geography, the spatial influence exhibits the characteristic of decaying with increasing distance, so we tend to use the inverse of the Euclidean distance between two cities to set the weights. In this paper, the formula for constructing the spatial weight matrix of geographic distance is constructed as follows:

$${\text{W}}_{\text{ij}}=\left\{\begin{array}{ll} 1/{\text{S}}_{\text{ij}}, & \text{i}\neq \text{j}\\ 0, & \text{i}=\text{j} \end{array}\right.$$

Where: W_ij_ is the distance spatial weight parameter between cities i and j; S_ij_ is the Euclidean distance between cities i and j.

#### (2) Economic distance matrix (W_e_)

However, the economic spatial interrelationship between the cells cannot be reflected by the distance alone. Therefore, we use the difference in GDP per capita as a measure to construct the economic distance matrix with the specific formula that:

$${\text{W}}_{\text{ij}}=\left\{\begin{array}{ll} 1/\left|{\text{X}}_{\text{i}}-{\text{X}}_{\text{j}}\right|, & \text{i}\neq \text{j}\\ 0, & \text{i}=\text{j} \end{array}\right.$$

Where: W_ij_ is the spatial weight parameter of economic distance between cities i, j; X_i_, X_j_ are the GDP per capita of cities i, j, respectively.

#### (3) Economic-geographic distance nested spatial matrix (W_ed_)

In fact, using only geographic distance and economic distance to measure the degree of spatial association still produces errors, because the economic development and closeness of connection of each unit is the result of multiple factors such as economic, cultural, and institutional. Therefore, we use the economic-geographic distance nested matrix to measure the differences in spatial linkages of each cell, with the formula:

$$\begin{array}{l} W_{ij}=W_{d}\times \text{diag}\left(\frac{{\bar{Y}}_{1}}{\bar{Y}},\frac{{\bar{Y}}_{2}}{\bar{Y}},\cdots ,\frac{{\bar{Y}}_{n}}{\bar{Y}}\right)\\ {\bar{Y}}_{i}=\frac{1}{t_{1}-t_{0}+1}\sum_{t=t_{0}}^{t_{1}}Y_{it},\bar{Y}=\frac{1}{n\left(t_{1}-t_{0}+1\right)}\sum_{i=t_{0}}^{t_{1}}\sum_{i=1}^{n}Y_{it} \end{array}$$

Where: W_ij_ is the spatial weight parameter of the nested economic-geographic distance between cities i, j; W_d_ is the constructed spatial weight matrix of geographic distance; ${\bar{Y}}_{i}$ is the average real GDP of city i in 2011–2018; $\bar{Y}$ represents the sum of the real GDP of cities in 2011–2018.

### 4.2 Coupled coordination degree models

Drawing on Wang and Kong et al. (2021 [28]), we use the modified coupling degree model, which is calculated as follows:

$$\text{C}=\sqrt{\left[\text{1-}\frac{\sum_{\text{i}>j,j=1}^{\text{n}}\sqrt{{\left({\text{U}}_{\text{i}}{\text{-U}}_{\text{j}}\right)}^{\text{2}}}}{\sum_{m=1}^{n-1}m}\right]*{\left(\prod_{i=1}^{n}\frac{{\text{U}}_{\text{i}}}{\text{max}{\text{U}}_{\text{i}}}\right)}^{\frac{1}{n-1}}}$$

$$\text{D}=\sqrt{\text{C}*\text{T}}$$

T=αf(x)+βg(y)

where C denotes the degree of coupling between subsystems, Ui and Uj denote the integrated evaluation value of digital economy and green economy respectively. D is the degree of coupling coordination, C is the degree of coupling, T is the degree of integrated coordination, f(x) and g(y) are the integrated evaluation value of digital economy and green development respectively, and α and β are the respective weights, where α and β are taken as the value of 0.5. According to the research of Yu and Liu and et al. [29] (2022) the degree of coupling coordination model is divided into five stages, respectively: Severe disorder(0.00<C≤0.20), Mild disorder(0.20<C≤0.40), Barely Coordinated(0.40<C≤0.60), Intermediate coordination(0.60<C≤0.80) and High Quality coordination(0.80<C≤1.00). As shown in Table 1.

**Table 1:** Stages of coupled coordination.

No.	Coupling coordination degree D	Phase
1	0.00<D≤0.20	Severe disorder
2	0.20<D≤0.40	Mild disorder
3	0.40<D≤0.60	Barely Coordinated
4	0.60<D≤0.80	Intermediate coordination
5	0.80<D≤1.00	High Quality coordination

### 4.3 Variable settings

#### 4.3.1 Independent variable

In this paper, the digital economy is chosen as the main explanatory variable. According to Tapscott (1996) [30], the term "digital economy" refers to an economic system that heavily utilizes ICT technologies, including infrastructure, e-commerce, and B2B, B2C, and C2C transaction models. The term "digital economy" was first established in a 1998 report by the U.S. Department of Commerce titled "The Emerging Digital Economy." To measure the scope and level of the digital economy, some academics have developed an accounting framework [31, 32]. However, many studies only use one variable to define the digital economy and do not offer a comprehensive measurement of the digital economy. Numerous studies have been conducted on the digital economy from the angles of digital industrialization as well as the digitalization of industries [33, 34]. Chinese research on the digital economy began later than studies from other countries, although the data sources are more varied. The level of development of the digital economy has been assessed by several academics at various scales, including national [9], provincial [35], city [36], district, and county [37].

At present, there is no unified standard for the evaluation index system of digital economy. Based on the previous definition of digital economy, this paper uses principal component analysis to analyze and measure, and finally constructs the digital economy evaluation index system from four dimensions: infrastructure, industrial development, innovation capability and financial transaction [5, 38].

Firstly, infrastructure is the foundation of the digital economy, the lack of infrastructure support digital economy will be difficult to develop. Referring to Luo et al. (2022) [39], infrastructure is measured by the number of Internet broadband access subscribers as well as the number of mobile phone subscribers. Second, the development of digital industry is a visual display of the flourishing digital economy, and the growth of the industry is inextricably linked to the development of telecommunication business, so the use of telecommunication business revenue to measure the development of the industry. Third, innovation capability is the core competitiveness of digital economy development. The digital economy, as a new economic form, needs innovation ability as support. Referring to Qian and Jiang (2020) [40], Zhang et al (2022) [41], the number of people in the information transmission computer services and software industry and the government’s scientific expenditure are used to measure the innovation capacity. Finally, the digital economy is developed by relying on the Internet, in which e-commerce, online payment and other means play an important role. Therefore, this paper takes digital finance as a measurement dimension of digital economy. And it introduces Peking University Digital Inclusive Finance Index [42] to measure the development level of digital finance. Subsequently, the entropy weighting method is used to assign weights to the indicators, and the comprehensive index of digital economy is finally calculated. Table 2 displays the precise indicators:

**Table 2:** Digital economy development evaluation index system.

Variable name	Variable interpretation	Mean	Variance
Infrastructure	Number of Internet broadband access subscribers	92.8593	113.311
	Number of cell phone subscribers	458.0863	494.3472
Industrial development	Revenue from telecommunication business	435883.9	728179.2
Innovation capability	Number of people in the information transmission computer services and software industry	12260.78	48307.34
	Government’s scientific expenditure	101262.3	334568
Financial transactions	Digital Inclusive Finance Index	155.9187	61.9673

#### 4.3.2 Dependent variable

In this paper, the green manufacturing efficiency of the considered non-desired output is used as the explanatory variable. In the measurement of green manufacturing efficiency, on the basis of measuring manufacturing efficiency from the input-output perspective, resource consumption and environmental pollution are also fully considered, so as to construct a green manufacturing total factor productivity index system that includes non-desired outputs. Among them, fixed asset input of the secondary industry, industrial electricity consumption, number of industrial employees, and built-up area are taken as each input factor of the manufacturing industry; industrial output is regarded as the output parameter; and emissions of industrial three wastes are taken as the non-desired output parameter of environmental pollution in the manufacturing industry. In this study, we adopt the perpetual inventory method to obtain the fixed asset stock of the secondary industry as fixed asset input of the secondary industry, based on the idea of Young (2003) [43], and obtain the real industrial output of each year by deflating the nominal industrial output of each year according to the price index [44], with 2011 as the base period.

minρ*=1+1m∑m=1Msmxxjmt1-1l+h∑l=1Lslyyjlt+∑h=1Hshbbjht,xjmt⩾∑j=1,j≠0nλjtxjmt+smxyjlt⩾∑j=1,j≠knλjtyjlt-slybjht⩾∑j=1,j≠knλjtbjht+shbλjt⩾0,smx⩾0,sly⩾0,j=1,⋯,n

where: ρ* is the green manufacturing efficiency value; $x_{j}^{t}$ denotes the input of j in period t; $y_{j}^{t}$, and $b_{j}^{t}$ are the desired output parameters and non-desired output parameters, respectively; m, l, and h denote the number of corresponding factors; $s_{m}^{x}、s_{l}^{y}、s_{h}^{b}$ denote the corresponding relaxation vectors; λ is the decision unit weight vector.

#### 4.3.3 Control variables

In the selection of control variables, drawing on previous studies, the level of economic development, the degree of industrial sophistication, the degree of government intervention, the level of openness to the outside world, innovation capability, and environmental regulation are selected as control variables for the relationship between the digital economy and green manufacturing efficiency. Where the level of economic development is measured using real per capita gdp. Industrial sophistication is measured using the ratio of value added in the secondary and tertiary sectors. The level of government intervention is measured using the share of government fiscal expenditure in gdp. The level of openness to the outside world is measured using the ratio of the number of foreign-invested enterprises to the total number of enterprises in the city. Innovation capacity is measured using the proportion of science and technology expenditure to fiscal expenditure. Finally, industrial SO2 removal, comprehensive utilization rate of solid waste, and smoke and dust removal are used as raw data to calculate the environmental regulation index using the entropy value method.

### 4.4 Data selection

This paper takes 2011 as the starting point of the study and uses panel data of 274 prefecture-level cities in China from 2011–2021 as the sample for the empirical study. The data used are obtained from the China Statistical Yearbook, the China Regional Statistical Yearbook, and the China City Statistical Yearbook. And the missing values of the panel data were filled by linear interpolation method.

## 5. Empirical analysis

### 5.1 The fundamental state of efficient green manufacturing

**Fig 2:**
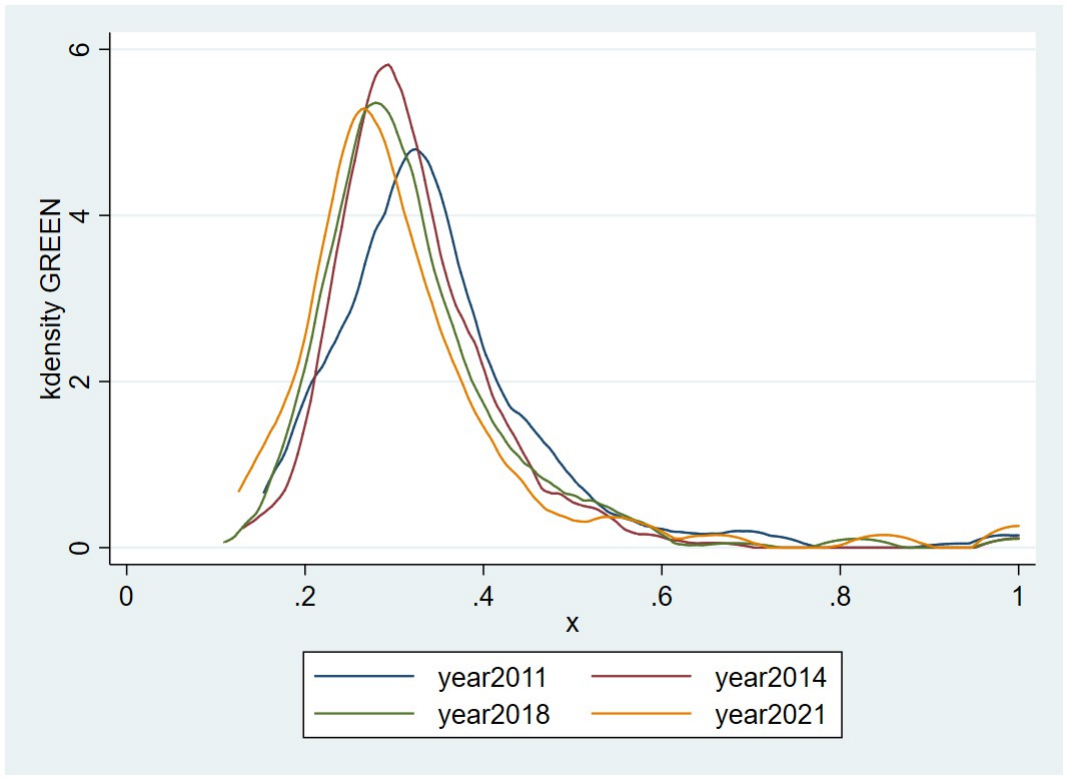
2011–2021 kernel density of green manufacturing efficiency in China.

As can be seen in Fig 2, from 2011 to 2021, the kernel density curve of green manufacturing efficiency in Chinese cities basically maintains stability, and its peak inflection points are all below the green manufacturing efficiency of 0.4, which implies that the overall green manufacturing level in China is low and at a low level. However, the curve shows a trend of leftward shift, indicating that the green manufacturing efficiency not only does not improve, but also shows a downward trend. The height of the wave peak is decreasing over time, indicating that China’s green manufacturing efficiency tends to be balanced and dynamically converged among regions. Therefore, it can be argued that from 2011 to 2021, the green manufacturing efficiency of Chinese cities has always been at a low level, and even shows a trend of decreasing rather than increasing, which is in urgent need of further development. However, on the other hand, the nationwide equilibrium has improved, and the gap between regions has narrowed, showing a dynamic convergence.

### 5.2 Regional differences in the coupling and coordination of digital economy and green manufacturing efficiency

By analysing the regional development of the digital economy and green manufacturing efficiency, it is found that there are relatively obvious regional differences in the degree of coupling and coordination between both systems. In Table 3, the coupling and coordination degree of digital economy and green manufacturing efficiency in seven regions of China is reflected, and it is found that there are relatively obvious differences between regions. It is mainly shown that the coupling coordination degree level is low in Southwest, North China and Northwest China, while the coupling coordination degree level is relatively high in East China, Central China and Northeast China. However, the overall trend shows a gradual decline, especially in 2017 and 2020 there is a clear downward inflection point. Therefore, it can be concluded that although there are obvious differences between regions, the differences are relatively small, and the coupling coordination degree of each region is at a low level overall, and shows a downward trend.

**Table 3:** Coupled coordination of digital economy and green manufacturing efficiency in seven regions of China.

	Northeast China	Southwest China	East China	North China	Central China	South China	Northwest China
2011	0.5142	0.4309	0.5666	0.4652	0.5396	0.5469	0.4444
2012	0.5084	0.4461	0.5704	0.4532	0.5459	0.5541	0.4502
2013	0.5189	0.4387	0.5472	0.4422	0.5357	0.5420	0.4522
2014	0.5146	0.4381	0.5469	0.4402	0.5280	0.5498	0.4381
2015	0.5353	0.4402	0.5424	0.4281	0.5239	0.5545	0.4340
2016	0.5231	0.4491	0.5566	0.4467	0.5392	0.5457	0.4317
2017	0.3643	0.3544	0.3727	0.3058	0.3303	0.4234	0.2965
2018	0.5186	0.4569	0.5502	0.4406	0.5247	0.5448	0.4271
2019	0.5119	0.4600	0.5965	0.4632	0.5916	0.6137	0.4628
2020	0.3275	0.3145	0.3727	0.2957	0.3227	0.3937	0.2911
2021	0.4536	0.4319	0.5641	0.4383	0.5188	0.5253	0.4059

### 5.3 Impact of digital economy on green manufacturing efficiency and spatial spillover effects

#### 5.3.1 Spatial correlation test

Before conducting the spatial econometric analysis, this paper first uses the global Moran’s I index to test the spatial correlation between regions. The specific calculation formula is as follows:

$$\text{I}=\frac{\sum_{i=1}^{n}\sum_{j=1}^{n}{\text{w}}_{ij}(x_{i}-\bar{x})(x_{j}-\bar{x})}{s^{2}\sum_{i=1}^{n}\sum_{j=1}^{n}{\text{w}}_{ij}}$$

where s^2^ is the sample variance and w_ij_ is the (i, j) element of the spatial weight matrix. The Moran index accepts values between [−1, 1], where [−1, 0] denotes negative correlation (high values next to low values) and [0, 1] denotes positive correlation (high values next to high values and low values next to low values). If it is equal to 0, there is no correlation, meaning that the objects are scattered randomly in space and there is no spatial link.

According to the test results, the green manufacturing efficiency likewise passed the significance test in all years with the exception of 2013–2015, and the digital economy also passed the significance test at the 1% level for Moran’s I in all years. It suggests that during the study period, there is a considerable spatial autocorrelation between the green manufacturing efficiency and the digital economy. Consequently, further research should be conducted utilizing the spatial econometric model. The results are shown in Table 4.

**Table 4:** Results of Moran’s I test for digital economy and green manufacturing efficiency from 2011–2021.

year	2011	2012	2013	2014	2015	2016	2017	2018	2019	2020	2021
DE	-0.044*** (-17.987)	-0.022*** (-8.157)	-0.037*** (-14.962)	-0.026*** (-9.922)	-0.026*** (-9.875)	-0.026*** (-10.147)	-0.027*** (-10.473)	-0.024*** (-9.072)	-0.022*** (-8.877)	-0.020*** (-8.025)	-0.019*** (-7.696)
GME	-0.010*** (-2.990)	-0.008* (-1.789)	-0.011*** (-3.230)	-0.006 (-1.034)	-0.005 (-0.005)	-0.004 (-0.273)	-0.008* (-1.818)	-0.011*** (-3.217)	-0.042*** (-18.923)	-0.014*** (-4.946)	-0.040*** (-18.095)

Note:

*, ** and *** are respectively significance level of 10%, 5% and 1%, and z values are in parentheses.

#### 5.3.2 Testing and selection of spatial econometric models

The spatial distribution properties of the variables were first tested by LM. Under the three weight matrices, LM-lag, LM-error, Robust LM-lag and Robust LM-error all passed the significance test at 1% statistical level, indicating that the constructed model should include both spatial lag effect and spatial error effect, and therefore the spatial Durbin model should be used for further analysis. Through LR test and wald test, it was found that both of them passed the significance test at 1% statistical level, indicating that the spatial Durbin model cannot degenerate into spatial lag model or spatial error model, so it is more reasonable to use the spatial Durbin model. Further, Hausman’s test is used to select the fixed-effects model and the random-effects model, and the test results obviously reject the original hypothesis, so the spatio-temporal dual fixed-effects spatial Durbin model should be selected. The results are shown in Table 5.

**Table 5:** Spatial econometric model test results.

Variables	*W*_*d*_	*W*_*e*_	*W*_*ed*_
*Moran’s I*	33.415***	8.897***	10.006***
*LM-error*	932.968***	76.581***	97.148***
*Robust LM-error*	719.283***	30.492***	60.807***
*LM-lag*	327.656***	52.368***	57.967***
*Robust LM-lag*	113.971***	6.279**	21.626***
*LR-lag*	40.30***	25.01***	46.14***
*LR-error*	39.33***	25.43***	41.49***
*Wald_spacial_lag*	60.31***	39.64***	46.65***
*Wald_spacial_error*	49.87***	39.03***	41.33***
*Hausman*	40.86***	60.30***	27.18***

Note:

*, ** and *** are respectively significance level of 10%, 5% and 1%

#### 5.3.3 Spatial econometric model regression results

In order to conduct the analysis of spatial spillover effect of digital economy on green manufacturing efficiency and obtain the results with robustness, this paper conducts SDM analysis based on spatial distance matrix, economic distance matrix, and nested matrix of economic and geospatial distance, respectively. Table 4 shows the estimation results of SDM under the three weight matrices. The results show that the impact of digital economy on green manufacturing efficiency passes the significance test at 1% statistical level under all three weight matrices. And the coefficients of all three are significantly positive, indicating that there is a significant contribution of the digital economy to green manufacturing efficiency. With the improvement of the level of digital economy, the green manufacturing efficiency will also be further improved. After considering the spatial lag term, the impact of digital economy on green manufacturing efficiency passes the significance test at the 5% statistical level under all three weighting matrices, and the coefficients of all three are significantly positive. It indicates that the enhancement of the level of digital economy will have a promoting effect on the green manufacturing efficiency in its neighbourhood. It means that the enhancement of the digital economy will have a spatial spillover effect on the green manufacturing efficiency of the neighbouring regions, and achieve the effect of common enhancement. In addition, the level of economic development, and environmental regulations have passed the significance test at 1% statistical level, and the coefficients are positive, indicating that there is a positive impact on green manufacturing efficiency. The coefficients of the advanced political industry and the degree of government intervention are significantly negative, indicating that there is a significant negative impact on green manufacturing efficiency. The results are shown in Table 6.

**Table 6:** Spatial Durbin model regression results.

Variables	*W*_*d*_	*W*_*e*_	*W*_*ed*_
*Digital economy*	0.0506***	0.0614***	0.0506***
	(0.0143)	(0.0142)	(0.0143)
*Economic development*	0.0462***	0.0485***	0.0462***
	(0.0174)	(0.0178)	(0.0174)
*Innovation capability*	0.0375**	-0.0223**	0.0375**
	(0.0169)	(0.0099)	(0.0169)
*Openness to the outside world*	-0.0160***	0.0383**	-0.0160
	(0.0106)	(0.0172)	(0.0106)
*Industrial sophistication*	-0.0067***	-0.0958***	-0.0067***
	(0.0017)	(0.0148)	(0.0017)
*Government intervention*	-0.1020***	-0.0063***	-0.1020***
	(0.0173)	(0.0019)	(0.0173)
*Environmental regulation*	0.0614***	0.0649***	0.0614***
	(0.0128)	(0.0130)	(0.0128)
*Wx* *Digital economy	0.0906**	0.0860**	0.0906**
	(0.0357)	(0.0332)	(0.0357)
*R* ^*2*^	0.0449	0.0449	0.0449
*Fixed time*	Yes	Yes	Yes
*Fixed space*	Yes	Yes	Yes
*N*	3014	3014	3014

Note:

*, ** and *** are respectively significance level of 10%, 5% and 1%, Standard errors are in parentheses

However, the SDM model’s estimate of the spatial interaction term of the digital economy does not completely capture the spatial impact of the digital economy on the effectiveness of green manufacturing. Therefore, using LeSage’s partial differential technique [45], it is required to divide the effects of the digital economy into direct effects and indirect effects. The direct effect of the digital economy on local green manufacturing efficiency and the indirect effect of the digital economy on the efficiency of green manufacturing in nearby regions are two of them. As a result, each of the three weight matrices is broken down and examined separately in this study. The precise outcomes are displayed in Table 7.

**Table 7:** Spatial Durbin model decomposition results.

Decomposition	Variables	*W*_*d*_	*W*_*e*_	*W*_*ed*_
**Direct effect**	*Digital economy*	0.0573***	0.0665***	0.0573***
		(0.0145)	(0.0147)	(0.0145)
	*Economic development*	0.0529***	0.0522***	0.0529***
		(0.0171)	(0.0171)	(0.0171)
	*Innovation capability*	0.0443***	-0.0242**	0.0443***
		(0.0167)	(0.0094)	(0.0167)
	*Openness to the outside world*	-0.0214**	0.0421**	-0.0214**
		(0.0101)	(0.0169)	(0.0101)
	*Government intervention*	-0.0107***	-0.0952***	-0.0107***
		(0.0039)	(0.0139)	(0.0039)
	*Industrial sophistication*	-0.1008***	-0.0074***	-0.1008***
		(0.0165)	(0.0028)	(0.0165)
	*Environmental regulation*	0.0630***	0.0653***	0.0630***
		(0.0134)	(0.0140)	(0.0134)
**Indirect effect**	*Digital economy*	0.1573***	0.1374***	0.1573***
		(0.0476)	(0.0422)	(0.0476)
	*Economic development*	0.1890**	0.1391**	0.1890**
		(0.0744)	(0.0590)	(0.0744)
	*Innovation capability*	0.1565**	-0.0885***	0.1565**
		(0.0646)	(0.0544)	(0.0646)
	*Openness to the outside world*	-0.1337***	0.1390	-0.1337***
		(0.0508)	(0.0491)	(0.0508)
	*Government intervention*	-0.1018	0.0124	-0.1018
		(0.0791)	(0.0455)	(0.0791)
	*Industrial sophistication*	0.0055	-0.0396	0.0055
		(0.0410)	(0.0438)	(0.0410)
	*Environmental regulation*	0.0424	0.0236	0.0424
		(0.0359)	(0.0348)	(0.0359)
**Total effect**	*Digital economy*	0.2146***	0.2038***	0.2146***
		(0.0494)	(0.0461)	(0.0494)
	*Economic development*	0.2419***	0.1913***	0.2419***
		(0.0796)	(0.0622	(0.0796)
	*Innovation capability*	0.2008***	-0.1127**	0.2008***
		(0.0712)	(0.0552)	(0.0712)
	*Openness to the outside world*	-0.1552***	0.1810***	-0.1552***
		(0.0518)	(0.0549)	(0.0518)
	*Government intervention*	-0.1125	-0.0828*	-0.1125
		(0.0826)	(0.0428)	(0.0826)
	*Industrial sophistication*	-0.0953**	-0.0470	-0.0953**
		(0.0378)	(0.0459)	(0.0378)
	*Environmental regulation*	0.1054**	0.0889**	0.1054**
		(0.0409)	(0.0417)	(0.0409)

Note:

*, ** and *** are respectively significance level of 10%, 5% and 1%, Standard errors are in parentheses

First, the impact of digital economy on local green manufacturing efficiency is analyzed by direct effects. Under the three weight matrices, the direct effects of digital economy on green manufacturing efficiency pass the significance test at 1% statistical level, and the coefficients are all positive. It indicates that there is a significant positive effect of digital economy on local green manufacturing efficiency, and as the level of digital economy increases, the local green manufacturing efficiency will also be significantly improved. In addition, the control variables of economic development level, government intervention level, environmental regulation and industry sophistication also pass the significance test. This indicates that the level of economic development, the degree of government intervention, environmental regulation and the advanced industry have a significant effect on the local green manufacturing efficiency.

Second, the impact of digital economy on green manufacturing efficiency in neighboring regions is analyzed through indirect effects. Under the three weight matrices, the indirect effect of digital economy on green manufacturing efficiency passes the significance test at 1%, and the coefficients are positive. It indicates that there is a significant positive effect of digital economy on green manufacturing efficiency in neighboring regions, which is the spillover effect. In addition, economic development level passes the significance test at all three weight matrices and the coefficients are positive. It indicates that there is a spatial spillover effect of economic development level on the green manufacturing efficiency of neighboring regions. With the improvement of economic development level, the green manufacturing efficiency of the neighboring regions will be improved accordingly.

### 5.4 Robustness analysis

To ensure the reliability of the results, this paper uses two methods for robustness testing. First, replacing the explanatory variables. Using digital infrastructure as a proxy variable for the digital economy and testing it using the SDM model. The results are shown in Table 6. Second, the samples are subjected to 1% (and 99%) two-sided tailing, and subsequently tested again using the SDM model using the tailed data. In both robustness tests, the spatial effect of the digital economy is significantly positive, the direct effect is significantly positive, and the indirect effect is significantly positive. This is consistent with the empirical analysis section above, indicating the robustness of the empirical results. The results are shown in Table 8.

**Table 8:** Robustness tests.

Decomposition	Variables	Substitution of explanatory variables			Tail reduction		
		*W*_*d*_	*W*_*e*_	*W*_*ed*_	*W*_*d*_	*W*_*e*_	*W*_*ed*_
**Main effect**	*Digital economy*	0.0288**	0.0363**	.0288**	0.0522***	0.0639***	0.0522***
		(0.0141)	(0.0149)	(0.0141)	(0.0183)	(0.0186)	(0.0183)
	Wx *Digital economy	0.1023**	0.0920*	0.1023**	0.0964**	0.0876**	0.0964**
		0.0474	(0.0471)	(0.0474)	(0.0378)	(0.0351)	(0.0378)
	Rho	0.3729***	0.3025***	0.3729***	0.3391***	0.2744***	0.3391***
		(0.0358)	(0.0378)	(0.0358)	(0.0363)	(0.0384)	(0.0363)
	Control variables	Yes	Yes	Yes	Yes	Yes	Yes
	*Fixed time*	Yes	Yes	Yes	Yes	Yes	Yes
	*Fixed space*	Yes	Yes	Yes	Yes	Yes	Yes
**Direct effect**	*Digital economy*	0.0364**	0.0419***	0.0364**	0.0592***	0.0691***	0.0592***
		(0.0142)	(0.0153)	(0.0142)	(0.0186)	(0.0191)	(0.0186)
	Control variables	Yes	Yes	Yes	Yes	Yes	Yes
	*Fixed time*	Yes	Yes	Yes	Yes	Yes	Yes
	*Fixed space*	Yes	Yes	Yes	Yes	Yes	Yes
**Indirect effect**	*Digital economy*	0.1714**	0.1419**	0.1716**	0.1637***	0.1384***	0.1637***
		(0.0688)	(0.0629)	(0.0688)	(0.0500)	(0.0444)	(0.0500)
	Control variables	Yes	Yes	Yes	Yes	Yes	Yes
	*Fixed time*	Yes	Yes	Yes	Yes	Yes	Yes
	*Fixed space*	Yes	Yes	Yes	Yes	Yes	Yes

Note:

*, ** and *** are respectively significance level of 10%, 5% and 1%, Standard errors are in parentheses

### 5.5 Intermediation effects analysis

In this paper, we will test the mediating effect of industrial agglomeration between digital economy and green manufacturing efficiency through Spatial Durbin’s mediating effect model (Wang & Guo, 2023). The specific modelling is as follows:

$$\begin{array}{l} {\text{Y}}_{\text{it}}=\alpha_{0}+\rho_{\text{1}}{\text{WY}}_{\text{it}}+\alpha_{\text{1}}{\text{A}}_{\text{it}}+\alpha_{\text{2}}{\text{WA}}_{\text{it}}+\alpha_{\text{3}}{\text{X}}_{\text{it}}+\alpha_{\text{4}}{\text{WX}}_{\text{it}}+\mu_{\text{i}}+\eta_{\text{t}}+\delta_{\text{it}}\\ {\text{M}}_{\text{it}}=\beta_{0}+\rho_{\text{2}}{\text{WM}}_{\text{it}}+\beta_{\text{1}}{\text{A}}_{\text{it}}+\beta_{\text{2}}{\text{WA}}_{\text{it}}+\beta_{\text{3}}{\text{X}}_{\text{it}}+\beta_{\text{4}}{\text{WX}}_{\text{it}}+\mu_{\text{i}}+\eta_{\text{t}}+\delta_{\text{it}}\\ {\text{Y}}_{\text{it}}=\gamma_{0}+\rho_{\text{3}}{\text{WY}}_{\text{it}}+\gamma_{\text{1}}{\text{A}}_{\text{it}}+\gamma_{\text{2}}{\text{WA}}_{\text{it}}+\gamma_{\text{3}}{\text{M}}_{\text{it}}+\gamma_{\text{4}}{\text{WM}}_{\text{it}}+\gamma_{\text{5}}{\text{X}}_{\text{it}}+\gamma_{\text{6}}{\text{WX}}_{\text{it}}+\mu_{\text{i}}+\eta_{\text{t}}+\delta_{\text{it}} \end{array}$$

Where Yit represents the explanatory variable green manufacturing efficiency, Ait denotes the explanatory variable digital economy, Mit represents the mediating variable and the level of industrial agglomeration, and Xit represents other control variables that will have an effect. μ denotes the province fixed effect, η denotes the time fixed effect, and δ denotes the random disturbance term.

**Fig 3:**
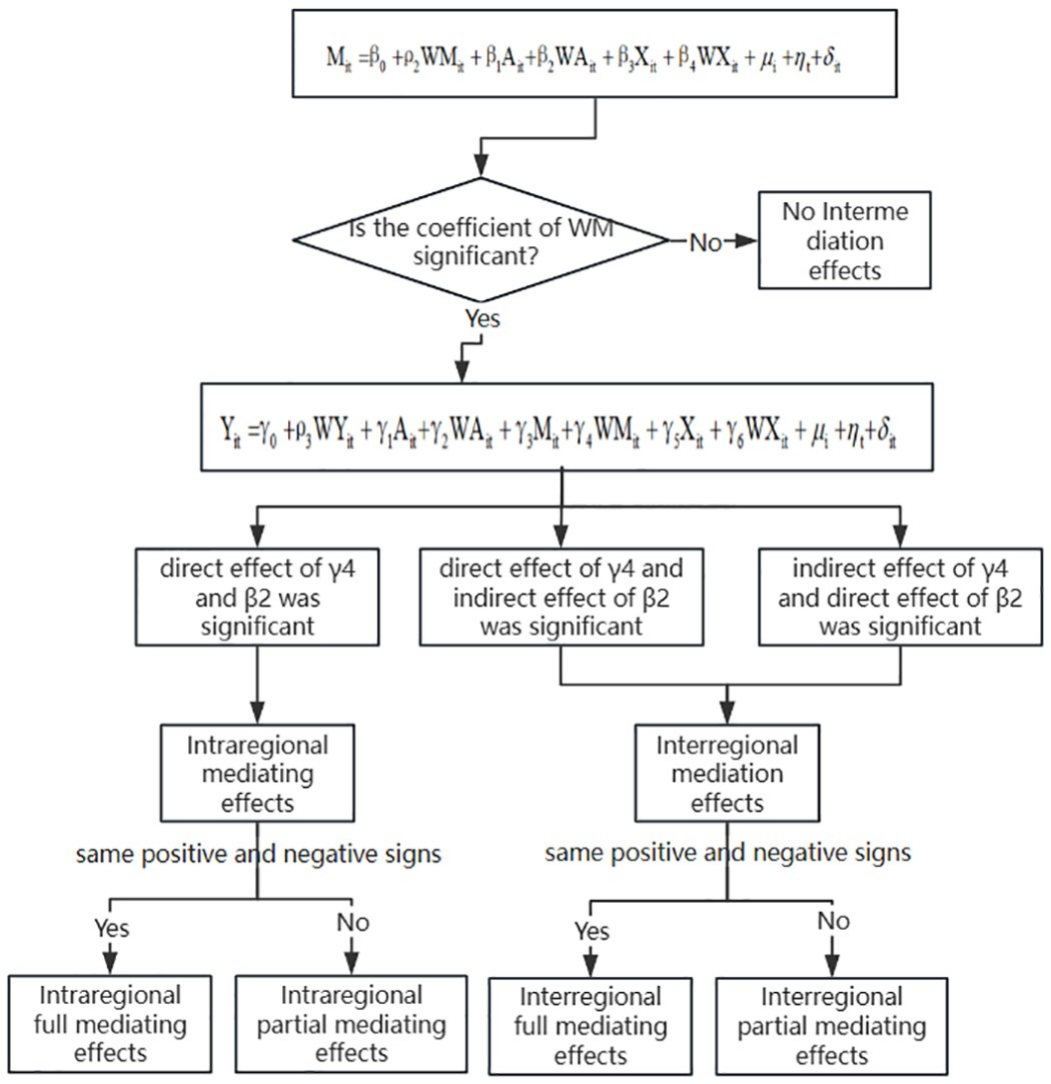
Model of the mechanism.

Referring to Zhang et al. (2023) [46], who used the location entropy index to calculate the degree of industrial agglomeration, the arithmetic mean of the degree of aggregation of secondary and tertiary industries is used to measure the level of industrial agglomeration in this study. The analytical model of the mechanism is shown in Fig 3. And the specific results are shown in Table 9.

**Table 9:** Mechanisms of influence testing.

		Model 1	Model 2	Model 3	Model 4
Decomposition	Variables	Industrial agglomeration	Green Manufacturing Efficiency	Industrial agglomeration	Green Manufacturing Efficiency
**Direct effect**	*Digital economy*	0.4056***	0.1185***	0.3007***	0.0838**
		(0.1259)	(0.0348)	(0.1109)	(0.0348)
	Industrial agglomeration	/	0.0232	/	0.0222
		/	(0.0146)	/	(0.0141)
	Control variables	Yes	Yes	Yes	Yes
	*Fixed time*	Yes	Yes	Yes	Yes
	*Fixed space*	Yes	Yes	Yes	Yes
**Indirect effect**	*Digital economy*	0.2395	0.2814***	0.4714***	0.4007***
		(0.1512)	(0.0848)	(0.1674)	(0.1095)
	Industrial agglomeration	/	0.0685*	/	0.0729**
		/	(0.0362)	/	(0.0357)
	Control variables	Yes	Yes	Yes	Yes
	*Fixed time*	Yes	Yes	Yes	Yes
	*Fixed space*	Yes	Yes	Yes	Yes

Note:

*, ** and *** are respectively significance level of 10%, 5% and 1%, Standard errors are in parentheses

In Table 9, Model 1 represents the effect of digital economy on the mechanism of industrial agglomeration. The results show that the direct effect of digital economy on industrial agglomeration passes the significance test at 1% statistical level and the coefficient is positive, while the indirect effect does not pass the significance test. Therefore, it can be assumed that the digital economy will significantly promote local industrial agglomeration, while the impact on the industrial agglomeration status of the neighbouring regions is not obvious. Model II represents the joint effect of digital economy and industrial agglomeration mechanism on green manufacturing efficiency. The results show that the direct effect of digital economy on green manufacturing efficiency passes the significance test at the 1% statistical level and the coefficient is positive after adding the industrial agglomeration mechanism. While industrial agglomeration did not pass the significance test. Therefore, it cannot be considered that industrial agglomeration plays an intra-regional mediating effect between digital economy and green manufacturing efficiency. By analysing the indirect effect, it is found that the indirect effect of digital economy on green manufacturing efficiency passes the significance test at 1% statistical level and the coefficient is positive. The indirect effect of industrial agglomeration on green manufacturing efficiency passes the significance test at the 1% statistical level, and the coefficient is positive. Therefore, it can be concluded that industrial agglomeration plays interregional mediating effect between digital economy and green manufacturing efficiency. Meanwhile, the coefficients of industrial agglomeration and digital economy have the same sign and are both positive, and according to the determination rule of mediation effect, industrial agglomeration plays a complete mediation effect.

Comprehensively, the above analysis found that there is a significant positive effect of digital economy on local industrial agglomeration, and there is a significant positive effect of industrial agglomeration on green manufacturing efficiency in the neighbouring regions, which ultimately leads to a positive effect of digital economy on green manufacturing efficiency in the neighbouring regions, in which the industrial agglomeration plays the inter-regional fully mediating effect. To ensure the reliability of the results, digital infrastructure is further used as a proxy variable for digital economy for robustness testing, and the results are shown in Model 3 and Model 4 in Table 9. It is consistent with the part of the empirical analysis above, indicating that the empirical results are robust.

## 6. Conclusions and recommendations

### 6.1 Conclusion and discussion

This paper empirically analyses the relationship between the digital economy and green manufacturing efficiency using panel data from 274 Chinese cities from 2011–2021, with a focus on the spatial correlation between the two. It is found that, first, the overall level of current green manufacturing efficiency is still low. And the overall level of coupling coordination degree is low, and there is an obvious regional gap, in which the eastern region is significantly higher than the western region. Second, the enhancement of the digital economy has an obvious promotion effect on the local green manufacturing efficiency. Third, there is an obvious spatial spillover effect of the digital economy on green manufacturing efficiency, i.e., the enhancement of the digital economy will promote the development of green manufacturing efficiency in the neighbouring regions, which presents a positive spatial spillover effect.

### 6.2 Recommendations

Based on the above research, this paper puts forward the following suggestions. First, accelerate the development of the digital economy to stimulate the new kinetic energy of urban green development. On the one hand, the government should focus on promoting digital technological innovation, establishing digital economy industrial bases, guiding enterprises to solve digital technological difficulties in green production, and promoting industrial enterprises to carry out green production. On the other hand, the government should also enhance the level of enterprises in various industries in the city in the fields of energy saving and environmental protection, clean production, etc., through increasing financial allocations for scientific research, reducing digital taxes and other financial policies, to achieve the development of green industry, and to improve the overall efficiency of green manufacturing in the city.

Second, implement differentiated development strategies, focusing on the leading role of green advanced cities. Attaching importance to the positive spatial spillover effect of the digital economy on the green manufacturing efficiency of the neighbouring regions, driving the later-developing cities with the first-developing cities, encouraging inter-regional exchanges and cooperation, and learning from each other’s advanced knowledge and experience. According to the boundary characteristics of the digital economy spillover, the layout of digital economy communication nodes at the junction of city clusters and urban agglomerations, to promote overall exchanges The government can establish cross-regional green development cooperation mechanisms and platforms to share experiences and technologies, carry out joint research and project cooperation, and ultimately achieve synergistic development of green manufacturing efficiency between regions.

Third, deepen industrial convergence and agglomeration, and smooth urban green development channels. Considering the important intermediary role played by industrial agglomeration, the government should start from its own construction and industrial adjustment to promote industrial convergence and agglomeration, and enhance the positive and active role of the digital economy in promoting green manufacturing efficiency. Specifically, the government should provide tax incentives, financial support, encourage inter-industry cooperation and other forms of industrial policies, encourage the industry to carry out integrated regulation and upstream and downstream cooperation, and achieve high-quality industrial synergistic agglomeration, thus strengthening its role in promoting the efficiency of the digital economy and green manufacturing.

### 6.3 Limitations and prospects

The research on the digital economy and efficient green manufacturing is enriched and supplemented to some extent by this publication. But there are some issues with this study. First, the statistics from 2011 to 2021 are selected because they are the most readily available. To guarantee the timeliness of the research, the years of data should be stretched further. Second, this study exclusively examines the linear link between digital economy and green manufacturing efficiency using a geographical panel model. The green development of manufacturing is, in reality, a dynamic process of change that is influenced by a variety of circumstances, and its interaction with the digital economy may exhibit a non-linear trend of change over time. For instance, during the early stages of the development of the digital economy, building infrastructure produces more carbon emissions, which causes green efficiency to decrease. The scale effect, which improves green efficiency, can be realized with the future development of the digital economy. In order to examine the link between the two, non-linear methods such panel threshold regression and generalized summation need be taken into account.
